# (*Z*)-3-(4-Chloro­phen­yl)-2-(2-phenyl­cyclo­hex-2-en-1-yl­imino)­thia­zolidin-4-one

**DOI:** 10.1107/S1600536812024294

**Published:** 2012-06-02

**Authors:** Chin Wei Ooi, Hoong-Kun Fun, Ching Kheng Quah, Murugan Sathishkumar, Alagusundaram Ponnuswamy

**Affiliations:** a X-ray Crystallography Unit, School of Physics, Universiti Sains Malaysia, 11800 USM, Penang, Malaysia; b Department of Organic Chemistry, School of Chemistry, Madurai Kamaraj University, Madurai 625 021, Tamil Nadu, India

## Abstract

The title compound, C_21_H_19_ClN_2_OS, exists in a *cis* conformation with respect to the N=C bond [1.2608 (13) Å]. The cyclo­hexene ring adopts a distorted half-chair conformation. The thia­zolidine ring is close to being planar (r.m.s. deviation = 0.057 Å) and makes dihedral angles of 62.92 (6) and 56.32 (6)°, respectively, with the benzene ring and the chloro-substituted benzene ring. The dihedral angle between the benzene ring and the chloro-substituted benzene ring is 72.91 (6)°. In the crystal, mol­ecules are linked by C—H⋯O and C—H⋯N hydrogen bonds into undulating sheets lying parallel to the *bc* plane. The crystal is further consolidated by C—H⋯π inter­actions.

## Related literature

For details of thia­zolidin-4-one derivatives, see: Previtera *et al.* (1994) ▶; Sharma *et al.* (2000 ▶); Kato *et al.* (1999*a* ▶,*b* ▶); Tanabe *et al.* (1991 ▶); Rawal *et al.* (2005 ▶); Voss *et al.* (2003 ▶). For related structures, see: Fun *et al.* (2011 ▶); Ooi *et al.* (2012 ▶). For ring conformations, see: Cremer & Pople (1975 ▶). For the stability of the temperature controller used in the data collection, see: Cosier & Glazer (1986 ▶).
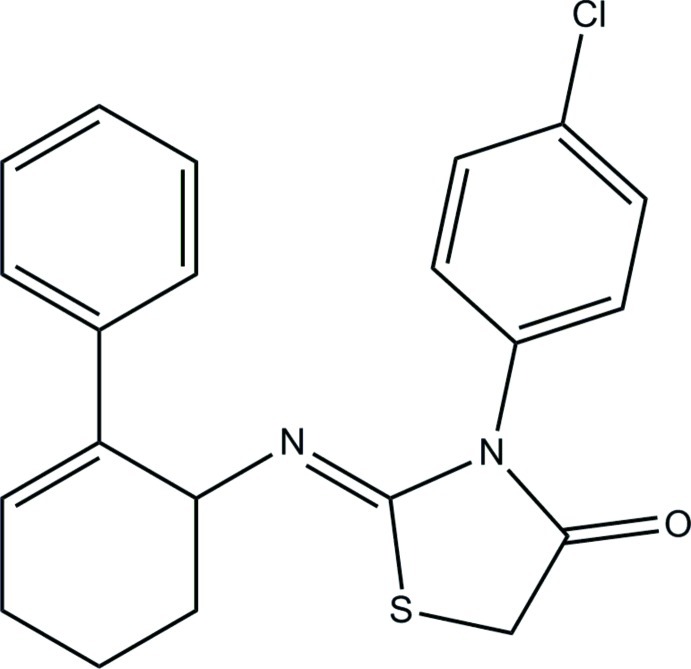


## Experimental

### 

#### Crystal data

C_21_H_19_ClN_2_OS
*M* *_r_* = 382.89
Monoclinic, 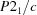

*a* = 9.1139 (3) Å
*b* = 17.4562 (6) Å
*c* = 12.9246 (4) Å
β = 118.640 (2)°
*V* = 1804.64 (10) Å^3^
*Z* = 4
Mo *K*α radiation
μ = 0.34 mm^−1^
*T* = 100 K
0.37 × 0.25 × 0.18 mm

#### Data collection

Bruker APEX DUO CCD diffractometer
Absorption correction: multi-scan (*SADABS*; Bruker, 2009 ▶) *T*_min_ = 0.886, *T*_max_ = 0.942
23788 measured reflections
5231 independent reflections
4605 reflections with *I* > 2σ(*I*)
*R*_int_ = 0.028

#### Refinement

*R*[*F* ^2^ > 2σ(*F* ^2^)] = 0.030
*wR*(*F* ^2^) = 0.083
*S* = 1.05
5231 reflections
235 parameters
H-atom parameters constrained
Δρ_max_ = 0.42 e Å^−3^
Δρ_min_ = −0.22 e Å^−3^

### 

Data collection: *APEX2* (Bruker, 2009 ▶); cell refinement: *SAINT* (Bruker, 2009 ▶); data reduction: *SAINT*; program(s) used to solve structure: *SHELXTL* (Sheldrick, 2008 ▶); program(s) used to refine structure: *SHELXTL*; molecular graphics: *SHELXTL*; software used to prepare material for publication: *SHELXTL* and *PLATON* (Spek, 2009 ▶).

## Supplementary Material

Crystal structure: contains datablock(s) global, I. DOI: 10.1107/S1600536812024294/hb6814sup1.cif

Structure factors: contains datablock(s) I. DOI: 10.1107/S1600536812024294/hb6814Isup2.hkl

Supplementary material file. DOI: 10.1107/S1600536812024294/hb6814Isup3.cml

Additional supplementary materials:  crystallographic information; 3D view; checkCIF report

## Figures and Tables

**Table 1:** Hydrogen-bond geometry (Å, °) *Cg*1 is the centroid of the C1–C6 benzene ring.

*D*—H⋯*A*	*D*—H	H⋯*A*	*D*⋯*A*	*D*—H⋯*A*
C10—H10*A*⋯O1^i^	0.99	2.53	3.4814 (17)	161
C11—H11*B*⋯O1^ii^	0.99	2.33	3.2411 (14)	152
C14—H14*B*⋯N1^iii^	0.99	2.62	3.4496 (14)	141
C17—H17*A*⋯*Cg*1^iv^	0.95	2.81	3.5913 (13)	140

Symmetry codes: (i) 
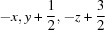
; (ii) 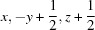; (iii) 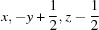; (iv) 
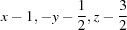
.

**Fig. 1.**
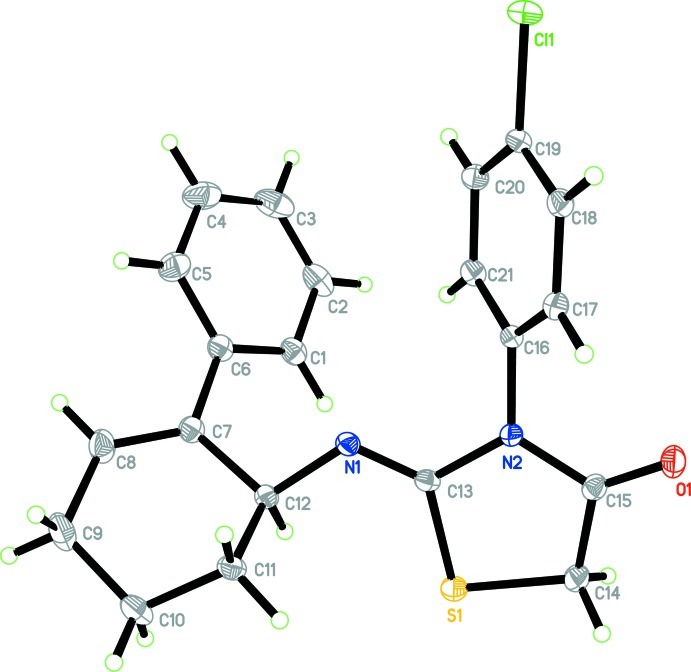
The molecular structure of the title compound, showing 50% probability displacement ellipsoids.

**Fig. 2.**
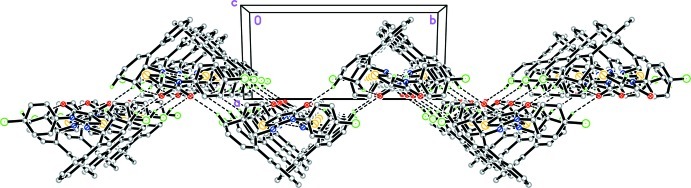
The crystal packing of the title compound, viewed along the c axis. H atoms not involved in the intermolecular interactions (dashed lines) have been omitted for clarity.

## supplementary crystallographic information

### Comment

Thiazolidin-4-one derivatives are known to exhibit diverse bioactivities such as anti-histaminic (Previtera *et al.*, 1994), anti-microbial (Sharma *et al.*, 2000), (Kato *et al.*, 1999*a*), PAF antagonist (Tanabe *et al.*, 1991), cardioprotective (Kato *et al.*, 1999*b*), anti HIV (Rawal *et al.*, 2005), and tumor necrosis factor-α antagonist activities (Voss *et al.*, 2003).

The title compound (Fig. 1) exists in *cis* configuration with respect to the N1 ═C13 bond [N1 ═C13 = 1.2608 (13) Å]. The cyclohexene (C7–C12) ring adopts a distorted sofa conformation and the puckering parameters are Q = 0.5004 (13) Å, θ = 130.94 (15)° and φ = 34.4 (2)° (Cremer & Pople, 1975). The thiazolidine (S1/N2/C13–C15) ring is essentially planar with a maximum deviation of 0.054 (1) Å at atom N2 and makes dihedral angles of 62.92 (6) and 56.32 (6)° respectively with the benzene ring (C1–C6) and chloro-substituted benzene ring (C16–C21). The dihedral angle between the benzene ring and chloro-substituted benzene ring is 72.91 (6)°. The bond lengths and angles are comparable to the related structures (Fun *et al.*, 2011 & Ooi *et al.*, 2012).

In the crystal structure (Fig. 2), molecules are linked *via* C10—H10A···O1, C11—H11B···O1 and C14—H14B···N1 hydrogen bonds (Table 1) into undulating sheets lying parallel to the *bc* plane. The crystal is further consolidated by C17—H17A···*Cg*1 interactions (Table 1), involving the centroid of the benzene ring (C1–C6; *Cg*1).

### Experimental

A mixture of 1-(4-chlorophenyl)-3-(2-phenylcyclohex-2-enyl)thiourea (0.5 g, 2.3 mmol) and chloroacetyl chloride (0.33 g, 4.6 mmol) was heated to reflux in 1,4-dioxane (10 ml) at 100 °C for 5 h. The reaction mixture was washed with diluted sodium bicarbonate solution (25 ml) and dried over anhydrous sodium sulfate. The solvent was then evaporated under reduced pressure and the resulting residue was subjected to column chromatography using silica gel (60–120 mesh) as the stationary phase and petroleum ether-ethyl acetate (90:10) as the mobile phase to give the pure product. Yield: 0.71 g (80%); *M.p.*: 156–157 °C. Colourless blocks were obtained by recrystallization from dichloromethane solution.

### Refinement

All the H atoms were positioned geometrically and refined using a riding model with *U*_iso_(H) = 1.2 *U*_eq_(C) (C—H = 0.95, 0.99 and 1.00 Å). In the final refinement, two outliers (1 1 7) and (-2 1 2) were omitted.

### Crystal data

**Table:**

C_21_H_19_ClN_2_OS	*F*(000) = 800
*M**_r_* = 382.89	*D*_x_ = 1.409 Mg m^−^^3^
Monoclinic, *P*2_1_/*c*	Mo *K*α radiation, λ = 0.71073 Å
Hall symbol: -P 2ybc	Cell parameters from 9952 reflections
*a* = 9.1139 (3) Å	θ = 2.6–34.3°
*b* = 17.4562 (6) Å	µ = 0.34 mm^−^^1^
*c* = 12.9246 (4) Å	*T* = 100 K
β = 118.640 (2)°	Block, colourless
*V* = 1804.64 (10) Å^3^	0.37 × 0.25 × 0.18 mm
*Z* = 4	

### Data collection

**Table:**

Bruker APEX DUO CCD diffractometer	5231 independent reflections
Radiation source: fine-focus sealed tube	4605 reflections with *I* > 2σ(*I*)
Graphite monochromator	*R*_int_ = 0.028
φ and ω scans	θ_max_ = 30.0°, θ_min_ = 2.1°
Absorption correction: multi-scan (*SADABS*; Bruker, 2009)	*h* = −12→12
*T*_min_ = 0.886, *T*_max_ = 0.942	*k* = −24→24
23788 measured reflections	*l* = −18→18

### Refinement

**Table:**

Refinement on *F*^2^	Primary atom site location: structure-invariant direct methods
Least-squares matrix: full	Secondary atom site location: difference Fourier map
*R*[*F*^2^ > 2σ(*F*^2^)] = 0.030	Hydrogen site location: inferred from neighbouring sites
*wR*(*F*^2^) = 0.083	H-atom parameters constrained
*S* = 1.05	*w* = 1/[σ^2^(*F*_o_^2^) + (0.0385*P*)^2^ + 0.767*P*] where *P* = (*F*_o_^2^ + 2*F*_c_^2^)/3
5231 reflections	(Δ/σ)_max_ = 0.002
235 parameters	Δρ_max_ = 0.42 e Å^−^^3^
0 restraints	Δρ_min_ = −0.22 e Å^−^^3^

### Special details

**Table:**

Experimental. The crystal was placed in the cold stream of an Oxford Cryosystems Cobra open-flow nitrogen cryostat (Cosier & Glazer, 1986) operating at 100 (1) K.
Geometry. All e.s.d.'s (except the e.s.d. in the dihedral angle between two l.s. planes) are estimated using the full covariance matrix. The cell e.s.d.'s are taken into account individually in the estimation of e.s.d.'s in distances, angles and torsion angles; correlations between e.s.d.'s in cell parameters are only used when they are defined by crystal symmetry. An approximate (isotropic) treatment of cell e.s.d.'s is used for estimating e.s.d.'s involving l.s. planes.
Refinement. Refinement of *F*^2^ against ALL reflections. The weighted *R*-factor *wR* and goodness of fit *S* are based on *F*^2^, conventional *R*-factors *R* are based on *F*, with *F* set to zero for negative *F*^2^. The threshold expression of *F*^2^ > σ(*F*^2^) is used only for calculating *R*-factors(gt) *etc*. and is not relevant to the choice of reflections for refinement. *R*-factors based on *F*^2^ are statistically about twice as large as those based on *F*, and *R*- factors based on ALL data will be even larger.

### Fractional atomic coordinates and isotropic or equivalent isotropic displacement parameters (Å^2^)

**Table:**

	*x*	*y*	*z*	*U*_iso_*/*U*_eq_	
Cl1	0.21570 (4)	−0.051642 (15)	1.02340 (2)	0.02160 (7)	
S1	0.24459 (4)	0.363135 (15)	0.69867 (2)	0.01731 (7)	
O1	0.04486 (10)	0.16687 (5)	0.56961 (7)	0.01895 (16)	
N1	0.27625 (11)	0.31077 (5)	0.90882 (7)	0.01307 (16)	
N2	0.18040 (11)	0.22633 (5)	0.74984 (7)	0.01253 (16)	
C1	0.63915 (13)	0.29044 (6)	1.06753 (10)	0.0170 (2)	
H1A	0.6105	0.3130	0.9933	0.020*	
C2	0.75211 (14)	0.22981 (7)	1.10844 (11)	0.0220 (2)	
H2A	0.7990	0.2111	1.0617	0.026*	
C3	0.79679 (15)	0.19637 (7)	1.21700 (12)	0.0277 (3)	
H3A	0.8734	0.1548	1.2444	0.033*	
C4	0.72833 (16)	0.22428 (8)	1.28510 (12)	0.0282 (3)	
H4A	0.7600	0.2024	1.3602	0.034*	
C5	0.61355 (15)	0.28412 (7)	1.24385 (10)	0.0219 (2)	
H5A	0.5658	0.3019	1.2906	0.026*	
C6	0.56708 (13)	0.31871 (6)	1.13445 (9)	0.01562 (19)	
C7	0.44377 (13)	0.38247 (6)	1.08949 (9)	0.01487 (19)	
C8	0.43293 (14)	0.43351 (7)	1.16306 (10)	0.0214 (2)	
H8A	0.5073	0.4276	1.2450	0.026*	
C9	0.31193 (16)	0.49927 (7)	1.12570 (11)	0.0255 (2)	
H9A	0.3714	0.5460	1.1689	0.031*	
H9B	0.2233	0.4879	1.1470	0.031*	
C10	0.23143 (15)	0.51445 (6)	0.99322 (11)	0.0214 (2)	
H10A	0.1324	0.5478	0.9685	0.026*	
H10B	0.3120	0.5411	0.9748	0.026*	
C11	0.17912 (13)	0.43895 (6)	0.92644 (9)	0.01592 (19)	
H11A	0.1197	0.4492	0.8406	0.019*	
H11B	0.1013	0.4118	0.9470	0.019*	
C12	0.33162 (13)	0.38816 (6)	0.95671 (9)	0.01352 (18)	
H12A	0.3971	0.4107	0.9204	0.016*	
C13	0.23863 (12)	0.29844 (6)	0.80285 (9)	0.01265 (18)	
C14	0.15339 (14)	0.29402 (6)	0.58042 (9)	0.0176 (2)	
H14A	0.0486	0.3146	0.5151	0.021*	
H14B	0.2318	0.2828	0.5496	0.021*	
C15	0.11748 (13)	0.22186 (6)	0.62889 (9)	0.01409 (18)	
C16	0.18581 (12)	0.16066 (6)	0.81802 (8)	0.01233 (18)	
C17	0.04100 (13)	0.11881 (6)	0.78834 (9)	0.01434 (18)	
H17A	−0.0632	0.1350	0.7254	0.017*	
C18	0.05075 (14)	0.05291 (6)	0.85211 (9)	0.01572 (19)	
H18A	−0.0464	0.0232	0.8324	0.019*	
C19	0.20399 (14)	0.03118 (6)	0.94472 (9)	0.01523 (19)	
C20	0.34831 (13)	0.07366 (6)	0.97650 (9)	0.01584 (19)	
H20A	0.4516	0.0586	1.0415	0.019*	
C21	0.33872 (13)	0.13850 (6)	0.91151 (9)	0.01426 (18)	
H21A	0.4364	0.1678	0.9308	0.017*	

### Atomic displacement parameters (Å^2^)

**Table:**

	*U*^11^	*U*^22^	*U*^33^	*U*^12^	*U*^13^	*U*^23^
Cl1	0.02893 (14)	0.01406 (12)	0.02091 (13)	0.00025 (10)	0.01122 (11)	0.00494 (9)
S1	0.02495 (14)	0.01306 (12)	0.01514 (12)	−0.00279 (9)	0.01059 (10)	0.00118 (9)
O1	0.0237 (4)	0.0184 (4)	0.0148 (3)	−0.0037 (3)	0.0093 (3)	−0.0040 (3)
N1	0.0136 (4)	0.0108 (4)	0.0129 (4)	−0.0009 (3)	0.0049 (3)	−0.0001 (3)
N2	0.0156 (4)	0.0107 (4)	0.0104 (4)	−0.0015 (3)	0.0054 (3)	0.0001 (3)
C1	0.0138 (4)	0.0152 (5)	0.0193 (5)	−0.0023 (4)	0.0058 (4)	−0.0026 (4)
C2	0.0135 (5)	0.0173 (5)	0.0327 (6)	−0.0014 (4)	0.0090 (4)	−0.0048 (4)
C3	0.0154 (5)	0.0196 (6)	0.0383 (7)	0.0030 (4)	0.0051 (5)	0.0053 (5)
C4	0.0227 (6)	0.0273 (6)	0.0253 (6)	0.0023 (5)	0.0041 (5)	0.0089 (5)
C5	0.0188 (5)	0.0248 (6)	0.0184 (5)	0.0007 (4)	0.0059 (4)	0.0025 (4)
C6	0.0122 (4)	0.0149 (5)	0.0157 (4)	−0.0018 (4)	0.0034 (4)	−0.0015 (4)
C7	0.0127 (4)	0.0149 (5)	0.0143 (4)	−0.0010 (4)	0.0043 (4)	−0.0012 (4)
C8	0.0194 (5)	0.0224 (5)	0.0171 (5)	0.0011 (4)	0.0045 (4)	−0.0055 (4)
C9	0.0251 (6)	0.0211 (6)	0.0260 (6)	0.0029 (5)	0.0088 (5)	−0.0091 (4)
C10	0.0212 (5)	0.0129 (5)	0.0277 (6)	0.0013 (4)	0.0096 (4)	−0.0018 (4)
C11	0.0156 (4)	0.0126 (4)	0.0164 (4)	0.0015 (4)	0.0050 (4)	0.0015 (4)
C12	0.0148 (4)	0.0107 (4)	0.0133 (4)	−0.0016 (3)	0.0053 (4)	−0.0008 (3)
C13	0.0126 (4)	0.0107 (4)	0.0139 (4)	−0.0001 (3)	0.0057 (3)	0.0013 (3)
C14	0.0244 (5)	0.0163 (5)	0.0137 (4)	−0.0018 (4)	0.0103 (4)	0.0002 (4)
C15	0.0150 (4)	0.0160 (5)	0.0124 (4)	0.0007 (4)	0.0075 (4)	−0.0002 (3)
C16	0.0154 (4)	0.0103 (4)	0.0113 (4)	0.0003 (3)	0.0064 (3)	−0.0002 (3)
C17	0.0145 (4)	0.0146 (4)	0.0122 (4)	−0.0008 (4)	0.0050 (3)	−0.0008 (3)
C18	0.0176 (5)	0.0144 (5)	0.0149 (4)	−0.0038 (4)	0.0076 (4)	−0.0019 (4)
C19	0.0222 (5)	0.0100 (4)	0.0143 (4)	0.0010 (4)	0.0093 (4)	0.0008 (3)
C20	0.0162 (4)	0.0142 (5)	0.0153 (4)	0.0034 (4)	0.0061 (4)	0.0008 (4)
C21	0.0133 (4)	0.0132 (4)	0.0156 (4)	−0.0001 (3)	0.0064 (4)	−0.0009 (3)

### Geometric parameters (Å, º)

**Table:**

Cl1—C19	1.7411 (11)	C8—H8A	0.9500
S1—C13	1.7780 (10)	C9—C10	1.5284 (18)
S1—C14	1.8067 (11)	C9—H9A	0.9900
O1—C15	1.2084 (13)	C9—H9B	0.9900
N1—C13	1.2608 (13)	C10—C11	1.5215 (15)
N1—C12	1.4707 (13)	C10—H10A	0.9900
N2—C15	1.3854 (12)	C10—H10B	0.9900
N2—C13	1.4096 (13)	C11—C12	1.5329 (14)
N2—C16	1.4322 (13)	C11—H11A	0.9900
C1—C2	1.3921 (15)	C11—H11B	0.9900
C1—C6	1.4027 (15)	C12—H12A	1.0000
C1—H1A	0.9500	C14—C15	1.5110 (15)
C2—C3	1.3883 (19)	C14—H14A	0.9900
C2—H2A	0.9500	C14—H14B	0.9900
C3—C4	1.388 (2)	C16—C17	1.3920 (14)
C3—H3A	0.9500	C16—C21	1.3920 (14)
C4—C5	1.3911 (17)	C17—C18	1.3929 (14)
C4—H4A	0.9500	C17—H17A	0.9500
C5—C6	1.4032 (15)	C18—C19	1.3873 (15)
C5—H5A	0.9500	C18—H18A	0.9500
C6—C7	1.4878 (15)	C19—C20	1.3895 (15)
C7—C8	1.3408 (15)	C20—C21	1.3871 (14)
C7—C12	1.5226 (14)	C20—H20A	0.9500
C8—C9	1.5021 (17)	C21—H21A	0.9500
			
C13—S1—C14	92.74 (5)	C10—C11—H11A	109.5
C13—N1—C12	118.28 (9)	C12—C11—H11A	109.5
C15—N2—C13	117.03 (8)	C10—C11—H11B	109.5
C15—N2—C16	121.51 (8)	C12—C11—H11B	109.5
C13—N2—C16	121.45 (8)	H11A—C11—H11B	108.0
C2—C1—C6	120.85 (11)	N1—C12—C7	108.91 (8)
C2—C1—H1A	119.6	N1—C12—C11	109.73 (8)
C6—C1—H1A	119.6	C7—C12—C11	111.23 (8)
C3—C2—C1	120.59 (11)	N1—C12—H12A	109.0
C3—C2—H2A	119.7	C7—C12—H12A	109.0
C1—C2—H2A	119.7	C11—C12—H12A	109.0
C2—C3—C4	119.33 (11)	N1—C13—N2	121.56 (9)
C2—C3—H3A	120.3	N1—C13—S1	128.54 (8)
C4—C3—H3A	120.3	N2—C13—S1	109.89 (7)
C3—C4—C5	120.29 (12)	C15—C14—S1	108.01 (7)
C3—C4—H4A	119.9	C15—C14—H14A	110.1
C5—C4—H4A	119.9	S1—C14—H14A	110.1
C4—C5—C6	121.17 (12)	C15—C14—H14B	110.1
C4—C5—H5A	119.4	S1—C14—H14B	110.1
C6—C5—H5A	119.4	H14A—C14—H14B	108.4
C1—C6—C5	117.75 (10)	O1—C15—N2	124.39 (10)
C1—C6—C7	120.77 (10)	O1—C15—C14	124.13 (9)
C5—C6—C7	121.48 (10)	N2—C15—C14	111.47 (9)
C8—C7—C6	121.25 (10)	C17—C16—C21	121.06 (9)
C8—C7—C12	121.21 (10)	C17—C16—N2	120.14 (9)
C6—C7—C12	117.54 (9)	C21—C16—N2	118.78 (9)
C7—C8—C9	124.89 (10)	C16—C17—C18	119.16 (9)
C7—C8—H8A	117.6	C16—C17—H17A	120.4
C9—C8—H8A	117.6	C18—C17—H17A	120.4
C8—C9—C10	112.15 (10)	C19—C18—C17	119.25 (10)
C8—C9—H9A	109.2	C19—C18—H18A	120.4
C10—C9—H9A	109.2	C17—C18—H18A	120.4
C8—C9—H9B	109.2	C18—C19—C20	121.87 (10)
C10—C9—H9B	109.2	C18—C19—Cl1	119.04 (8)
H9A—C9—H9B	107.9	C20—C19—Cl1	119.09 (8)
C11—C10—C9	109.69 (10)	C21—C20—C19	118.72 (10)
C11—C10—H10A	109.7	C21—C20—H20A	120.6
C9—C10—H10A	109.7	C19—C20—H20A	120.6
C11—C10—H10B	109.7	C20—C21—C16	119.91 (10)
C9—C10—H10B	109.7	C20—C21—H21A	120.0
H10A—C10—H10B	108.2	C16—C21—H21A	120.0
C10—C11—C12	110.92 (9)		
			
C6—C1—C2—C3	−0.49 (17)	C15—N2—C13—N1	170.00 (9)
C1—C2—C3—C4	−0.36 (18)	C16—N2—C13—N1	−9.89 (15)
C2—C3—C4—C5	1.30 (19)	C15—N2—C13—S1	−9.15 (11)
C3—C4—C5—C6	−1.42 (19)	C16—N2—C13—S1	170.96 (7)
C2—C1—C6—C5	0.38 (16)	C14—S1—C13—N1	−175.13 (10)
C2—C1—C6—C7	−178.76 (10)	C14—S1—C13—N2	3.95 (8)
C4—C5—C6—C1	0.56 (17)	C13—S1—C14—C15	1.41 (8)
C4—C5—C6—C7	179.69 (11)	C13—N2—C15—O1	−170.49 (10)
C1—C6—C7—C8	−147.73 (11)	C16—N2—C15—O1	9.40 (16)
C5—C6—C7—C8	33.17 (16)	C13—N2—C15—C14	10.39 (13)
C1—C6—C7—C12	31.62 (14)	C16—N2—C15—C14	−169.72 (9)
C5—C6—C7—C12	−147.49 (10)	S1—C14—C15—O1	174.28 (9)
C6—C7—C8—C9	−179.63 (11)	S1—C14—C15—N2	−6.60 (11)
C12—C7—C8—C9	1.05 (18)	C15—N2—C16—C17	−54.52 (13)
C7—C8—C9—C10	−14.39 (18)	C13—N2—C16—C17	125.37 (10)
C8—C9—C10—C11	44.28 (14)	C15—N2—C16—C21	123.55 (10)
C9—C10—C11—C12	−63.22 (12)	C13—N2—C16—C21	−56.56 (13)
C13—N1—C12—C7	−153.55 (9)	C21—C16—C17—C18	−1.28 (15)
C13—N1—C12—C11	84.48 (11)	N2—C16—C17—C18	176.74 (9)
C8—C7—C12—N1	−139.34 (11)	C16—C17—C18—C19	0.93 (15)
C6—C7—C12—N1	41.32 (12)	C17—C18—C19—C20	0.53 (16)
C8—C7—C12—C11	−18.28 (14)	C17—C18—C19—Cl1	−179.52 (8)
C6—C7—C12—C11	162.38 (9)	C18—C19—C20—C21	−1.63 (16)
C10—C11—C12—N1	169.61 (9)	Cl1—C19—C20—C21	178.41 (8)
C10—C11—C12—C7	49.04 (12)	C19—C20—C21—C16	1.27 (15)
C12—N1—C13—N2	−178.32 (9)	C17—C16—C21—C20	0.16 (15)
C12—N1—C13—S1	0.66 (14)	N2—C16—C21—C20	−177.89 (9)

### Hydrogen-bond geometry (Å, º)

Cg1 is the centroid of the C1–C6 benzene ring.

**Table:**

*D*—H···*A*	*D*—H	H···*A*	*D*···*A*	*D*—H···*A*
C10—H10*A*···O1^i^	0.99	2.53	3.4814 (17)	161
C11—H11*B*···O1^ii^	0.99	2.33	3.2411 (14)	152
C14—H14*B*···N1^iii^	0.99	2.62	3.4496 (14)	141
C17—H17*A*···*Cg*1^iv^	0.95	2.81	3.5913 (13)	140

Symmetry codes: (i) −*x*, *y*+1/2, −*z*+3/2; (ii) *x*, −*y*+1/2, *z*+1/2; (iii) *x*, −*y*+1/2, *z*−1/2; (iv) *x*−1, −*y*−1/2, *z*−3/2.
